# Corporate Social Responsibility and Collective OCB: A Social Identification Perspective

**DOI:** 10.3389/fpsyg.2019.02720

**Published:** 2019-12-11

**Authors:** Xiao-Hua (Frank) Wang, Jun Yang, Rujiao Cao, Byron Y. Lee

**Affiliations:** ^1^Beijing Key Laboratory of Applied Experimental Psychology, National Demonstration Center for Experimental Psychology Education, Faculty of Psychology, Beijing Normal University, Beijing, China; ^2^Department of Management, University of North Carolina at Greensboro, Greensboro, NC, United States; ^3^Department of Management & Organization, University of Maryland, College Park, College Park, MD, United States; ^4^China Europe International Business School, Shanghai, China

**Keywords:** CSR, collective OCB, organizational prestige, collective organizational identification, sequential mediation

## Abstract

Although plenty of evidence has shown a positive relationship between collective organizational citizenship behavior (OCB) and unit or organizational performance, the antecedents of collective OCB are still understudied. In this study, we identify corporate social responsibility (CSR) as a new antecedent of firm-level collective OCB. Furthermore, we develop a collective social identification approach to examining the mechanism through which CSR may enhance collective OCB. Specifically, we propose and test a sequential mediation model in which CSR promotes organizational prestige. Organizational prestige, in turn, increases employees’ collective organizational identification and, consequently, enhances their collective OCB at the firm level. To test this model, we collected data from three different sources (i.e., HR director, CEO, and employees) from 160 firms in China. The results supported the hypotheses.

## Introduction

*Organizational citizenship behavior (OCB)* is defined as “individual behavior that is discretionary, not directly or explicitly recognized by the formal reward system, and that in aggregate promotes the effective functioning of the organization” (Organ, 1988, p. 4). Examples of OCB include helping others at work, taking on extra assignments, and promoting and protecting the organization (Bolino and Turnley, 2003). Amabile et al. (2014) suggests that in top-performing companies, it is a norm that employees display OCB in their daily work, such as helping and supporting each other, in order to do the best work possible. According to Cross et al. (2016), the time spent by managers and employees in OCB behaviors has increased by more than 50% over the past two decades. Although OCB has received extensive attention from management researchers, most research has studied OCB at the *individual level* (Organ et al., 2005). An important assumption of the OCB literature is that OCB on the collective level serves to provide additional critical resources for the organization, thereby improving the effectiveness of the organization as a whole (Organ, 2018). *Collective OCB* is defined as the extent to which employees of an organization collectively engage in OCB (Gong et al., 2010; Chun et al., 2013). Indeed, the literature demonstrates a positive effect of collective OCB on organizational-level outcomes (e.g., productivity, efficiency, reduced costs) (Podsakoff et al., 2009).

Although OCB is expected to benefit the originations in its aggregation (Organ, 2018), most studies examining the antecedents of OCB have focused on the individual level (Podsakoff et al., 2017; Ocampo et al., 2018), leaving the antecedents of collective OCB largely understudied. Recently, using data from more than 300 organizations, Cross et al. (2016) found that the distribution of OCB is often extremely lopsided, with 20 to 30% of OCB coming from 3 to 5% of employees. This finding highlights the importance of studying the antecedents of collective OCB. That is, organizations would benefit much more from collective OCB (i.e., most of the employees engaging in OCB together) rather than lopsidedly distributed individual OCB (i.e., only a few top performers engaging in OCB).

Since collective OCB is defined as a collective phenomenon, drivers of collective OCB must be conceptualized and measured at the collective level as well (Klein et al., 1994). Previous studies have reported that top management’s ethical leadership (Shin, 2012) and a high-performance work system (Sun et al., 2007; Gong et al., 2010) are firm-level antecedents of collective OCB. Drawing on social identity theory (Tajfel and Turner, 1986; Ashforth and Mael, 1989), our study extends this line of research by focusing on *corporate social responsibility (CSR)* as another important antecedent of collective OCB. CSR refers to a company’s discretionary actions and policies that appear to advance social well-being beyond its immediate financial interest and legal requirements (McWilliams and Siegel, 2001).

The CSR literature has been dominated by the macro-level perspective, in which business strategy scholars strive to reveal the relationships between firm-level CSR initiatives and indicators of firm performance, such as corporate financial performance (Glavas, 2016; Kim et al., 2018). Macro-level research has advanced our understanding of CSR by highlighting how firms’ CSR practices are shaped by the broader economic, political, and societal contexts (Marquis and Qian, 2013; Qian et al., 2015). Recently, however, CSR scholars have started to adopt an micro-level perspective, focusing their attention on how CSR practices may influence the company’s own people — that is, the employees (Morgeson et al., 2013; Jones et al., 2019). Micro-level CSR research is of theoretical significance because employees are important stakeholders who both influence and are influenced by their employer’s CSR initiatives (Aguinis and Glavas, 2019). Although evidence has supported the positive effect of CSR on employees’ OCB, all those studies were conducted at the individual level (Lin et al., 2010; Hansen et al., 2011; Ong et al., 2018). Thus, we have yet to know how CSR may influence employees’ OCB at the firm level. We argue that it is of theoretical importance to explicitly examine the relationship between CSR and collective OCB at the firm level, because relationships observed at the individual level may not hold at the firm level (Ostroff, 1992, 1993).

As OCB is expected to benefit the organization through its aggregation, our understanding of CSR’s impacts on OCB would not be complete without considering whether and how CSR may relate to employees’ OCB at the firm level. To this end, our study attempts to investigate the association between CSR practices and firm-level collective OCB.

**FIGURE 1:**
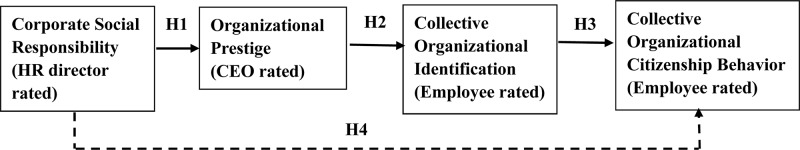
Research model.

In addition, we advance a theoretical model that elucidates the mechanism through which CSR may impact employees’ collective OCB at the firm level. Drawing on social identity theory (Ashforth and Mael, 1989), we propose that perceived external prestige and employees’ collective organizational identification will mediate, in a sequential manner (i.e., three-path mediation model), the relationship between CSR and collective OCB. First, CSR practices will be positively related to *organizational prestige*, defined as employees’ belief about how other people outside the organization, such as customers, competitors, and suppliers, judge or evaluate the status and prestige of the organization (Ashforth and Mael, 1989). Second, organizational prestige is expected to be positively related to employees’ *collective organizational identification (OI)*, defined as the extent to which employees hold a shared feeling of attachment and belonging to their organization (Van Der Vegt and Bunderson, 2005). Third, collective OI is expected to be positively related to employees’ collective OCB. Figure 1 depicts our research model.

This study makes several theoretical contributions to the literature. First, most prior research on the antecedents of OCB has been conducted at the individual level (Podsakoff et al., 2017). As OCB is expected to benefit the organization in its aggregation (Organ, 2018), it is of theoretical importance to examine the firm-level factors that may impact employees’ OCB as a whole (Shin, 2012). Thus, our work contributes to the OCB literature by identifying CSR practices as a new firm-level antecedent of collective OCB.

Second, previous studies on the antecedents of collective OCB (Gong et al., 2010; Shin, 2012) have mainly drawn on social exchange theory (Blau, 1964; Cropanzano et al., 2017). That is, employees are expected to engage in collective OCB in return for the kind treatment they receive from the organization—for example, in the form of ethical leadership (Shin, 2012) or a high-performance work system (Gong et al., 2010). Our study advances an alternative mechanism. We apply social identity theory (Ashforth and Mael, 1989) to the collective level and propose that CSR may influence collective OCB by shaping the company’s organizational prestige and employees’ collective OI in a sequential manner. Following prior studies (Van Der Vegt and Bunderson, 2005; Kearney et al., 2009), we consider collective OI as an emergent state and define it as the emotional significance that employees attach to their membership in the organization. We argue that the collective social identity perspective can serve as an alternative framework to explain how collective OCB emerges within the organization. Different from social exchange theory (Cropanzano et al., 2017) which treats employees and the organization as exchange partners, social identity theory posits that employees tend to rely on the status or social standing of their organization to evaluate their own self-worth (Ashforth and Mael, 1989). Tajfel (1972) first introduced the concept of social identity as “the individual’s knowledge that he belongs to certain social groups together with some emotional and value significance to him if the group membership” (p. 31). Social identity theory argues that people tend to classify themselves and others into various social categories, such as organizational membership, religious affiliation, gender, and age cohort (Tajfel and Turner, 1986). Categories are defined by prototypical characteristics abstracted from the members (Tajfel and Turner, 1986). This classification serves two important functions: individuals use such information to define themselves as well as other people in the social environment. In defining themselves, individuals tend to use information from social classification to reduce the ambiguity about who they are. In addition, they also use the categorization information and seek memberships in groups that enhance their self-image and self-value (Turner et al., 1979). According to this theory, employees are likely to identity with organizations perceived to be prestigious, because doing so can enhance their self-worth and fulfill their need for self-enhancement (Farooq et al., 2017).

Third, the macro-level and micro-level CSR studies undertaken in the past have often been fragmented (Chun et al., 2013). Most macro-level research has focused only on the relationship between firm-level CSR practices and corporate financial performance (Wang and Qian, 2011; Kim et al., 2018), but we still know very little regarding the effects of firm-level CSR on employees’ *collective* attitudes and behaviors (Jones et al., 2017). Although micro-level research has revealed how employees’ perceptions of CSR initiatives may influence their attitudes and behavior (Farooq et al., 2017; Wang et al., 2017; Rupp et al., 2018), this line of research more commonly focuses on the individual level of analysis only. Recently, Jones et al. (2017) urged researchers to explore how firm-level CSR may influence employees’ reactions *as a whole*, because employees’ collective reactions and behaviors may help explain why CSR practices are beneficial for other firm-level outcomes (Bauman and Skitka, 2012). Answering this call, our study attempts to bridge the macro- and micro-level perspectives by exploring the mechanism through which firm-level CSR practices may promotive employees’ collective OCB.

### Theoretical Background and Hypotheses

#### Corporate Social Responsibility

Previous macro-level CSR studies have largely focused on the impact of CSR practices on external stakeholders. That is, CSR practices can help increase a firm’s performance by improving the firm’s reputation, legitimacy, and relationships with external stakeholders, such as customers, suppliers, and vendors (Hosmer, 1994; Jones, 1995). Recently, researchers have sought to investigate how CSR may influence internal stakeholders (i.e., the employees) (Glavas, 2016; Gond et al., 2017; Jones et al., 2017), because a firm’s operational and financial performance also depend largely on its employees (Combs et al., 2006). Specifically, previous studies have reported the positive effects of CSR on individual employees’ OCB (Hansen et al., 2011; Ong et al., 2018).

This line of research has two notable limitations. First, almost all those studies (Hansen et al., 2011; Ong et al., 2018; Tian and Robertson, 2019) were conducted at the individual level and focus on how individual employees’ perceptions of CSR may influence their individual identification with the organization and their own OCB. Firm-level collective OCB, however, is not simply the sum of individual behaviors (Ehrhart and Naumann, 2004). Becker et al. (1997) contend that employees’ attitudes and behaviors in their aggregation play a pivotal role in creating sustained competitive advantages for the organization. As yet, we still have yet to know how CSR practice may influence employees’ attitudes and behaviors as a whole.

Second, many of the prior studies (Farooq et al., 2017; van Dick et al., in press) are subject to methodological problems—specifically, common method variance—because the independent, mediating, and dependent variables were all reported by the same source, the employees. This research design may result in inflation of relationships (Podsakoff et al., 2003).

To address these two limitations, our study conceptualizes CSR practice at the firm level, and proposes that CSR may influence employees’ firm-level collective OCB by enhancing organizational prestige and employees’ collective OI. We overcome the methodological problems of previous studies due to common method variance by collecting data from three different sources [i.e., human resources (HRs) director, chief executive officer (CEO), and employees]. A firm-level investigation of CSR’s impact on employees with a robust research design is of theoretical importance because it reveals the extent to which a firm’s CSR practice can enhance its competitive advantage by shaping employees’ attitudes and behaviors as a whole.

#### CSR and the Company’s Organizational Prestige

*Organizational prestige* is defined as employees’ beliefs about how other people outside the organization, such as customers, competitors, and suppliers, view or evaluate their organization (Ashforth and Mael, 1989; Dutton et al., 1994). It “is based on the employee’s evaluation of the extent to which organizational outsiders hold the firm in high regard or esteem because of the positive, socially valued characteristics of the organization” (Fuller et al., 2006, p. 819). Decades ago, Simon and March (1958) contended that prestige reflects the organization’s societal position, which is determined by the extent to which the organization possesses those attributes or characteristics recognized and valued by the society.

We propose that CSR acts as an important antecedent of organizational prestige for the following reasons. First, CSR promotes the organization’s visibility to the public (Simon and March, 1958). External stakeholders form their evaluations of the organization based on the informational cues or signals they receive directly from the organization or indirectly from the media. Organizations usually take great efforts to ensure that their engagements in community development and philanthropy are visible to external stakeholders (Bhattacharya and Sen, 2004). Such efforts are often recognized and valued by the communities that are the direct beneficiaries of the CSR initiatives. As a result, organizations’ CSR actions help them obtain respect and trust from the communities (Brammer and Pavelin, 2006). Indeed, CSR has been identified as a strategic investment for companies to build and maintain favorable relationships with their focal communities (McWilliams et al., 2006).

Second, an organization’s engagement in CSR may have a positive impact on other stakeholders’ (e.g., government, customers, suppliers) judgments about that organization, which also influence its prestige (Fombrun and Shanley, 1990). A firm’s prestige reflects public opinions about the firm, which largely depend on the extent to which the firm’s actions and behaviors are in accordance with stakeholders’ expectations (Brammer and Pavelin, 2006). Nowadays, stakeholders expect organizations to not only pursue economic or financial outcomes, but also demonstrate social benefits and advance social welfare. In turn, organizations that actively engage in CSR initiatives are more likely to enjoy higher prestige because those activities satisfy stakeholders’ expectations (Galbreath and Shum, 2012). Indeed, a firm’s participation in community development and philanthropy has been found to enhance outsiders’ perceptions of the firm (Brammer and Millington, 2005; Hsu, 2012).

Third, although CSR practices mainly focus on external stakeholders, they can also influence organizational prestige as perceived by the internal employees. This relationship arises because employees seek information about how outsiders view or evaluate their organization through word of mouth and public media (Smidts et al., 2001). Employees tend to compare the distinctive and charitable practices of their own organization with those of other firms and develop their perceptions of organizational prestige (Dutton et al., 1994). Thus, we propose CSR practices will be positively related to organizational prestige because external stakeholders are not only the targets of CSR initiatives but also important determinants of employees’ prestige judgments.

Hypothesis 1: CSR will be positively related to organizational prestige.

#### Organizational Prestige and Collective Organizational Identification

We propose that organizational prestige will be positively related to employees’ *collective organizational identification*, which is defined as the extent to which employees hold a shared feeling of attachment and belonging to their organization (Van Der Vegt and Bunderson, 2005). Although OI was originally conceptualized as an individual-level construct, social identity theory (Hogg and Terry, 2000) recognizes the fact that it is often a collectively shared state for two reasons. First, many antecedents of OI, such as organizational distinctiveness and intergroup competition (Tajfel and Turner, 1986; Ashforth and Mael, 1989), may have shared influences on all employees within the same organization. Second, through constant communication and interactions, employees in an organization may mutually reinforce one another’s individual OI and converge into a shared collective state (Morgeson and Hofmann, 1999). Following prior studies (Kearney et al., 2009; Dietz et al., 2015), we conceptualize OI at the collective level and propose that it will be influenced by organizational prestige.

According to social identity theory, organizational prestige is one of the most important antecedents of OI (Ashforth and Mael, 1989; Dutton et al., 1994). First, individuals tend to identify with a social group with high prestige because doing so can enhance their own self-concept (Tajfel and Turner, 1986). Similarly, employees desire to identify with organizations with high social standing or status so as to enhance their own self-worth and fulfill their need for self-enhancement (Dutton et al., 1994). Being a member of an organization that has positive and socially respected characteristics makes employees believe that they also hold those positive and socially respected characteristics (Fuller et al., 2006). Therefore, employees who identify with prestigious organizations obtain a corresponding sense of personal status (Simon and March, 1958).

Second, organizational prestige may promote employees’ OI by creating a sense of belongness for the employees (Mignonac et al., 2018). Research has shown that employees tend to hold prosocial values and expect those values to be practiced in the organizations they work for Jones et al. (2014). When employees perceive their company as possessing higher organizational prestige, they will feel that outsiders believe their organization demonstrates a strong commitment to important social values, such as contributing to the community and preserving the natural environment. Thus, employees may perceive that the values of the organization are aligned with their personal values (Carmeli et al., 2007). Such a feeling of value congruence may create a sense of belongness and motivate employees to identify more strongly with the organization (Brammer et al., 2015). Prior studies have supported the positive relationship between organizational prestige or image and OI at the individual level (Fuller et al., 2006; Farooq et al., 2014).

The previously mentioned individual responses to organizational prestige form the foundation for understanding collective OI. As stated earlier, collective OI emerges from employees’ common exposure to organizational prestige and their ongoing social interactions (Morgeson and Hofmann, 1999). As a result, when an organization accrues positive prestige, its employees may eventually develop a high level of collective OI.

Hypothesis 2: Organizational prestige will be positively related to employees’ collective organizational identification.

#### Collective Organizational Identification and Collective OCB

*Collective OCB* is defined as the extent to which employees of an organization collectively engage in OCB (Gong et al., 2010; Chun et al., 2013). Collective OCB derives from individual-level OCB, in that this firm-level phenomenon encompasses a firm’s employees aggregate OCB and reflects interpersonal dynamics among organizational members (Shin, 2012). Although OCB was initially conceptualized as an individual-level construct, recently scholars have called for more research on collective OCB because it is employees’ collective engagement in OCB, rather than their individual OCB, that promotes the effective functioning of the organization (Podsakoff et al., 2009; Organ, 2018).

The OCB literature contends that firm-level collective OCB may emerge through two processes. First, the social information processing perspective (Salancik and Pfeffer, 1978) posits that individuals utilize information and cues surrounding them to construct their perception of the reality. Employees working in the same firm are exposed to the same organizational factors, such as organizational culture, top management leadership, and CSR practices. As a result, they are likely to develop a shared understanding with regard to the normative level of their OCB (Ehrhart and Naumann, 2004). For instance, when the majority of the employees volunteer for overtime when needed, shared norms and expectations for this OCB emerges. Second, firm-level collective OCB is impacted by attraction–selection–attribution processes (Schneider, 1987). That is, organizations that expect their employees to display a high level of OCB tend to attract and hire individuals who are willing to engage in such behavior. Those employees who do not fit into such an environment will eventually leave the organization, resulting a homogenous level of OCB within the firm.

We further argue that employees’ collective OI will be positively related to their collective OCB for the following reasons. First, social identity theory suggests that employees, when they identify with the organization, regard the goals of the firm as intrinsically motivating and are likely to exhibit self-sacrificial and organization-oriented behaviors (Dutton et al., 1994). Employees with high OI tend to perceive their organizational successes as their personal successes. Collectively, employees who share high levels of OI have stronger commitment to organization goals and, therefore, are more likely to devote greater effort to their tasks and display higher levels of OCB (Ellemers et al., 2004; Wang and Howell, 2012).

Second, when employees identify with an organization that practices CSR, these employees are more likely to activate and reinforce their self-images as altruistic and helpful (Jones, 2010). This alignment occurs because their sense of self is consistent with the organization’s goals and values. Therefore, the employees are motivated to display higher levels of collective OCB, as this behavior will help them maintain their social identity as typical members of the organization (Oakes et al., 1991).

Indeed, substantial evidence has shown that, at the individual level, OI is positively related to OCB (Van Dick et al., 2006; Jones, 2010; Wang and Howell, 2012). We argue that when a high level of OI is shared among employees in an organization, this collective OI may shape the patterns of interactions among employees and generate normative expectations for their OCB.

Hypothesis 3: Employees’ collective organizational identification will be positively related to their collective OCB.

#### Mediating Effects of Organizational Prestige and Collective Organizational Identification

Given the theory and empirical evidence just presented, we propose that organizational prestige and collective OI may sequentially mediate the link between CSR and collective OCB. First, drawing on social identity theory (Ashforth and Mael, 1989), we propose that organizational prestige may mediate the positive impact of firm-level CSR on employees’ collective OI (Fuller et al., 2006). As stated above, firm-level CSR is expected to be positively related to organizational prestige for three reasons: (a) CSR practices promote the organization’s visibility to the public, and hence help the organization obtain respect and trust from the communities (Brammer and Pavelin, 2006); (b) CSR initiatives result in positive evaluations from external stakeholders (e.g., government and customers) because those initiatives satisfy those stakeholders’ expectations for modern organizations to advance social welfare (Galbreath and Shum, 2012); (c) those external stakeholders’ positive evaluation and judgment about the focal organization will also boost the internal employees’ perceived organizational prestige (Dutton et al., 1994). Organizational prestige, in turn, is expected to boost employees’ collective OI for the following two reasons: (a) identifying with a prestigious organization can enhance their self-worth and fulfill their need for self-enhancement (Ashforth and Mael, 1989); (b) organizational prestige may enhance employees’ collective OI by creating a sense of belongness for the employees (Farooq et al., 2014).

Second, collective OI is expected to mediate the positive effect of organizational prestige on employees’ collective OCB (Jones, 2010; Wang et al., 2017). Collective OI may have a positive impact on collective OCB for the following reasons: (a) Employees with high OI perceive their organizational successes as their personal successes, and therefore tend to exhibit self-sacrificial and organization-oriented behaviors (Wang and Howell, 2012); (b) employees, who identify with an organization that practices CSR, are more likely to activate and reinforce their self-images as altruistic and helpful, and thus are motivated to display higher levels of collective OCB (Jones, 2010). Consequently, we propose that CSR is related to collective OCB first through organizational prestige, and then through employees’ collective OI.

Hypothesis 4: Organizational prestige and collective organizational identification will sequentially mediate the positive relationship between CSR and employees’ collective OCB.

## Materials and Methods

### Data and Sample

The data for this study were drawn from a large-scale survey in China that collected multisource data from the CEO, the HR director, and multiple employees in each firm. To recruit respondents, we first searched a list of alumni of a major university in China and identified potential participants who were CEOs or senior executives in their company. We gave senior executives time to consult with their CEO in the company about the research project before giving us the final decision. After each company confirmed its participation, we asked the HR department to randomly select five employees from each of three departments in the company (e.g., R&D, marking, and production). We sent a standardized email to the HR director of each company, which emphasized the importance of random sampling and provided detailed instructions regarding how to randomly select employees to participate our study. Specifically, the HR director was told to randomly select five employees from the roster list. Management professors and trained graduate students visited each individual company and verbally confirmed with the HR staff that all participants were selected randomly. Then, they distributed the questionnaires, and were available to answer any questions that arose during the survey process.

All participants were assured that their participation was voluntary and that their responses would be anonymous and confidential. They had the right not to answer any question when they did not feel comfortable providing an honest answer. To reduce evaluation concerns and response biases, it was also emphasized that there were no correct or incorrect answers for any item in the survey. The CEOs reported the CSR activities and prestige of their company. The HR directors reported their company’s CSR activities as well. To avoid common method bias (Podsakoff et al., 2003), we used HR director-rated CSR in our hypotheses testing. As stated above, a random sample of 15 full-time employees within each company were invited to report their OI and OCB.

We have followed incentive approach suggested by Dillman (2011) and reimbursed all participants with a 50 RMB (equals to about $7.70 US dollars) prepaid phone card. Of the 256 sampled companies, 183 agreed to participate in the study (response rate = 71.5%). The incentive plan partly contributed to the satisfactory response rate in our study. This response rate is also in the reasonable range of mail surveys (57–71%, Messer and Dillman, 2011). The initial data set consisted of 2035 full-time employees and their corresponding CEOs and HR directors from 183 firms. Eighteen firms were removed from subsequent data analyses due to missing data in our key variables of interest (i.e., 10 firms had missing data on HR-rated CSR and eight firms had missing data on CEO-rated prestige), the remaining sample consisted of 1833 employees and their corresponding HR directors and CEOs from 165 firms. Additionally, as discussed later, five firms that demonstrated poor psychometric properties in aggregating employee responses to the firm level were removed. This resulted in the final sample of 1784 employees from 160 firms. We have run Little’s MCAR test and found that Missing data were MCAR (missing completely at random), and the results are χ^2^ = 311.02, df = 293, *p* = 0.22.

The median organizational size of the companies in the final sample was 170 employees, with an average of 11 employees representing each company. The final sample included 48% women and the participants had an average organizational tenure of 5 years (*SD* = 5.3). In terms of age, 45.6% of participants were younger than age 30; 37.9% were between 31 and 40; 13.9% were between 41 and 50; and 2.5% were older than age 51. As for their education, 3.6% held a middle school diploma, 19.9% a high school diploma, 36.3% a college degree, 34.8% a bachelor’s degree, and 5.4% a master’s degree or higher.

### Measures

All questions in the survey were in Chinese because this was the first and primary language for all respondents in this study. We followed the standard back-translation methodology (Brislin, 1970) to translate all the items to Chinese. All variables were measured using a five-point Likert scale, ranging from 1 (“Strongly disagree”) to 5 (“Strongly agree”).

Corporate social responsibility and organizational prestige were measured at the firm level, whereas collective OI and collective OCB were measured at the individual level. To justify the aggregation of employee responses on OI and OCB to the firm level, *r*_wg(j)_, ICC(1), and ICC(2) were assessed (Bliese, 2000). Five firms had *r*_wg(j)_ scores lower than 0.50 for either of the two individual-level variables. Thus, the employees from those 5 firms were removed from the sample, leading to the final sample of 160 firms.

#### Corporate Social Responsibility

Both the CEOs and HR directors of the firms reported their company’s CSR initiatives using five items adopted from Lichtenstein et al. (2004) and Kim et al. (2010). Sample items are “This organization is committed to using a portion of its profits to help non-profit organizations” and “This organization gives back to the communities in which it does business.” We collected data on CSR from two sources so that we could cross-validate our results. We used the HR directors’ ratings (α = 0.91) in hypotheses testing to avoid common method bias (Podsakoff et al., 2003). When we reran the analyses with CEO-reported CSR (α = 0.90), all the results remained unchanged.

#### Organizational Prestige

Organizational prestige was rated by the CEOs using a six-item scale (α = 0.85) adapted from Mael and Ashforth (1992) and Carmeli et al. (2006). The CEO reported his/her belief about how outside stakeholders (e.g., customers, competitors) view or perceive the prestige of his/her firm. Sample items are “Our customers consider our company as one of the best” and “Our competitors think highly of our company.”

#### Collective Organizational Identification

We used two items from Mael and Ashforth (1992) to measure organizational identification at the individual level (α = 0.67). A sample item is “When someone says anything bad about my company, I feel it personally.” In the analyses, mean *r*_wg(j)_ = 0.85, ICC (1) = 0.31, ICC (2) = 0.83, suggesting a sufficient level of agreement in employee responses (LeBreton and Senter, 2008). Thus, we aggregated the employees’ OI scores to the firm level.

#### Collective OCB

We used five items from Podsakoff et al. (1990) to measure collective OCB at the individual level (α = 0.86). Following Gong et al. (2010), we adopted a referent shift consensus model and changed the references of the items from “I” to “we.” A sample item reads, “We are willing to help each other on unit-relevant tasks.” In the analyses, mean *r*_wg(j)_ = 0.91, ICC(1) = 0.27, ICC(2) = 0.81, suggesting a sufficient level of agreement in employee responses. Thus, we aggregated the employees’ OCB scores to the firm level.

#### Control Variables

Following Gong et al. (2010), we controlled for firm size and ownership. Firm size was indicated by the number of employees reported by the HR directors. We dummy coded ownership into six groups: state owned, joint venture, privately owned, collectively owned, wholly foreign owned, and unknown ownership. Unknown ownership was set as the default category. However, the results showed that all the coefficients involving the control variables were non-significant, and the statistical significance of the findings was identical with or without the control variables in the model. According to Becker (2005) and Spector and Brannick (2011), including non-significant control variables is unnecessary and even undesirable in analyses because it may reduce statistical power or distort the relationships among the main variables. Thus, in the hypotheses testing, no control variable was included, and the results without the control variables were reported.

## Results

We first conducted a confirmatory factor analysis at the individual level, so as to assess the discriminant validity of the two employee-reported variables: OI and collective OCB. The two-factor model fit the data better [χ^2^(12) = 114.57, *p* < 0.01; RMSEA = 0.07, SRMR = 0.02, CFI = 0.98, TLI = 0.97] than the one-factor model did [χ^2^(14) = 703.60, *p* < 0.01; RMSEA = 0.17, SRMR = 0.07, CFI = 0.87, TLI = 0.80]. These results support the empirical distinctiveness of the two employee-reported variables.

Table 1 presents the descriptive statistics and correlations among all the variables at the firm level.

**TABLE 1:** Means, standardized deviations, and interrelations.

**Variables**	***M***	***SD***	**1**	**2**	**3**	**4**	**5**	**6**	**7**	**8**	**9**
(1) Firm size	1462.59	8394.01									
(2) Ownership dummy: state owned	0.07	0.25	0.44^∗∗^								
(3) Ownership dummy: joint venture	0.10	0.30	–0.04	–0.09							
(4) Ownership dummy: privately owned	0.72	0.45	–0.22^∗∗^	–0.43^∗∗^	–0.53^∗∗^						
(5) Ownership dummy: collectively owned	0.04	0.21	–0.01	–0.06	–0.07	–0.34^∗∗^					
(6) Ownership dummy: wholly foreign owned	0.04	0.19	0.01	–0.05	–0.07	–0.32^∗∗^	–0.04				
(7) CSR^a^	3.37	0.94	0.06	–0.02	0.01	0.01	0.06	−0.05			
(8) Organizational prestige	3.85	0.67	−0.16^∗^	–0.05	–0.06	0.08	–0.02	−0.05	0.28^∗∗^		
(9) Collective OI^b^	3.68	0.44	0.03	0.04	−0.20^∗^	0.14	0.01	−0.09	0.24^∗∗^	0.36^∗∗^	
(10) Collective OCB^b^	3.61	0.41	0.06	0.01	–0.14	0.10	0.03	−0.10	0.41^∗∗^	0.33^∗∗^	0.65^∗∗^

**N* = 160. ^*a*^CSR = corporate social responsibility; ^*b*^individual-level variables were aggregated to the firm level. ^∗^*p* < 0.05; ^∗∗^*p* < 0.01. Two-tailed.*

Following the recommendations of Preacher et al. (2010), we used Multilevel SEM to with Mplus to test our hypotheses, which generated unbiased estimates of the between-group effects at the firm level. Hypothesis 1 predicted that CSR would be positively related to organizational prestige. As shown in Model 1 in Table 2, CSR was positively related to organizational prestige (*r* = 0.22, *p* < 0.01), supporting H1.

**TABLE 2:** Regression results of CSR on collective OCB.

	**Organizational prestige**	**Collective OI**		**Collective OCB**	
	**M1**	**M2**	**M3**	**M4**	**M5**
**Independent variables**					
CSR^a^	0.22^∗∗^ (0.06)	0.11^∗∗^ (0.04)	0.07 (0.04)	0.18^∗∗^ (0.03)	0.11^∗∗^ (0.02)
Organizational prestige			0.21^∗∗^ (0.05)	0.02 (0.04)	0.01 (0.04)
Collective OI					0.61^∗∗^ (0.10)

**N* = 160; standard errors are presented in brackets. ^*a*^CSR = corporate social responsibility. ^∗∗^*p* < 0.01.*

Hypothesis 2 predicted that organizational prestige would be positively related to employees’ collective OI. As shown in Model 3 in Table 2, organizational prestige was significantly related to collective OI (*r* = 0.21, *p* < 0.01), after controlling for CSR. Thus, H2 was supported.

Hypothesis 3 proposed that collective OI would be positively related to collective OCB. As shown in Model 5 in Table 2, collective OI was significantly related to collective OCB (*r* = 0.61, *p* < 0.01), after controlling for CSR and organizational prestige. Thus, H3 was supported.

Hypothesis 4 predicted that organizational prestige and collective OI would sequentially mediate the positive relationship between CSR and collective OCB. We tested the three-path mediation model using the PROCESS program developed by Hayes (2017). The bootstrapping results indicated that the three-path mediation effect (CSR–organizational prestige–collective OI–collective OCB) was positive and significant [indirect effect = 0.02; 95% confidence interval = 0.01, 0.05]. This result indicated that organizational prestige and collective OI sequentially mediated the relationship between CSR and collective OCB, providing support for H4. After controlling for the effects of organizational prestige and collective OI, the relationship between CSR and collective OCB was still significant (*r* = 0.11, *p* < 0.01; see Model 5 in Table 2), suggesting that organizational prestige and collective OI only partially mediated the relationship between CSR and collective OCB.

## Discussion

This study proposes CSR as an innovative antecedent of collective OCB and explores how and why CSR may promote firm-level collective OCB. The results clearly support the proposed theoretical model. That is, CSR had a positive effect on organizational prestige, which in turn had a positive effect on collective OI. Collective OI was positively related to collective OCB. Furthermore, organizational prestige and collective OI sequentially mediated the positive relationship between CSR and collective OCB.

### Theoretical Contributions

Our study offers theoretical contributions to several different literatures. First, although OCB has received much attention in the literature, the antecedents of collective OCB are understudied. Several studies (Hansen et al., 2011; van Dick et al., in press) have suggested that, at the individual level, employees’ perception of their company’s CSR activities is positively related to their OCB, but we still do not know to what extent CSR may influence employee OCB as a whole. Thus, our study contributes to the OCB literature by identifying CSR as a firm-level antecedent of collective OCB. Specifically, our results indicate that, at the firm level, CSR practices may enhance employees’ collective OCB by promoting organizational prestige and collective OI.

Second, we contribute to social identity theory by extending OI to the firm level and exploring its mediating role in the relationship between CSR and collective OCB. Although OI was originally conceptualized as an individual-level construct, it may emerge as a shared collective state due to employees’ exposure to similar contextual variables and their ongoing interactions with one another (Morgeson and Hofmann, 1999). Indeed, researchers have started to examine the effects of collective OI in team processes. For example, Van Der Vegt and Bunderson (2005) found that collective team identification moderated the relationship between expertise diversity and team learning behavior and team performance reported that collective team identification moderated the indirect effect of objective diversity on team performance via team learning and team efficacy, such that the indirect effect was significant only when collective team identification was high. Although these studies have advanced our understanding of the role of identification at the collective level, most of them focused on the moderating role of collective identification. It is still not clear which factors may contribute to the emergence of collective identification and how collective identification, once emerged, may directly impact collective outcomes. In attempting to fill this gap, our study reveals that firm-level CSR may enhance employees’ collective OI by promoting organizational prestige, and that collective OI in turn has a positive effect on employees’ collective OCB. To our knowledge, our study is one of the first to explicitly investigate the mediating role of collective OI. In doing so, it takes an initial step toward developing a collective social identification perspective linking CSR, organizational prestige, collective OI, and collective OCB.

Third, we contribute to the CSR literature by bridging the macro-level and micro-level perspectives of CSR studies. Specifically, our study builds on the micro-level CSR studies that have recognized the mediation role of OI in the relationship between employees’ perception of CSR and OCB at the individual level (Jones, 2010; Farooq et al., 2017). Extending this line of research to the firm level, our study overcame the problems of common method bias by collecting data from three different sources. Our results suggest that, at the firm level, an organization’s CSR practices can promote collective OCB by enhancing its organizational prestige and employees’ collective OI. As collective OCB has been found to be positively related to firm performance (Chun et al., 2013), our findings shed light on how a firm’s CSR practice may enhance its competitive advantages by shaping its employees’ collective attitudes and behaviors as a whole.

### Practical Implications

Our study also has important practical implications. Top management of organizations should be aware that their CSR practices may influence both external and internal stakeholders simultaneously. First, CSR may help creating competitive advantages for the organization by boosting its external prestige. External stakeholders tend to believe that more socially responsible companies are more credible and trustworthy, and thus perceive these companies more favorably in terms of prestige and reputation. By making their CSR initiatives visible to the public, organizations may receive positive regard and enjoy a more favorable reputation with key external stakeholders, such as customers and competitors. Therefore, organizations should actively engage in corporate communications about their CSR activities via advertisements, promotions and CSR reports, which can help the organization build a positive image among external stakeholders.

Second, our results suggested that CSR practices may also influence internal stakeholders—that is, employees—by promoting their collective OI and subsequently their collective OCB. A deeper understanding of how employees may respond to CSR can enable organizations to better serve employees, communities and the society. Employee attitudes and behaviors are scarce, unique, and valuable resources for all organizations. Our findings indicate that CSR may change internal employees’ behavior via enhanced identification. Thus, CSR assists maintain a more industrial and loyal workforce, which in turn may benefit business performance of the company. If top management wishes to foster greater collective OI and collective OCB among the firm’s employees, an effective strategy could be actively communicating the positive impacts of CSR activities to those employees and help them understand enhanced external prestige resulted from those CSR practices. Taken together, our findings provide good reasons for organizations to adopt social responsibility as a firm-level strategy to gain competitive advantages.

### Limitations and Future Research

This study has several limitations that call for future research. First, following previous studies (Gong et al., 2010; Hooshangi and Loewenstein, 2016), we recruited participants from alumni of one university in China. Although our participants came from a variety of different industries (e.g., agriculture, manufacturing, telecommunication, etc.), our data were not perfectly representative of all industries, which may limit the generatability of our results. We encourage future research to test our model using a more representative data sample.

Second, we asked employees to self-report their collective OCB. We recognize that the best source of ratings for OCB remains a topic of debate. Although it is not uncommon to use a self-report measure of OCB (Farooq et al., 2017; van Dick et al., in press) because employees are usually the best observers of their own extra-role behaviors at work, we encourage future studies to cross-validate our results by collecting other-rated (e.g., supervisor or peer) OCB.

Third, we built our theoretical model based on social identity theory and, therefore, identify organizational prestige and collective OI as the mediators. However, the partial mediation results indicated that additional mediators might potentially explain the linkage between CSR and collective OCB. For example, Ong et al. (2018) recently found that employees’ prosocial motivation serves as an alternative mediator between CSR and OCB at the individual level. In addition, when Hu and Liden (2015) aggregated prosocial motivation to the team level, they found that team prosocial motivation had positive impacts on a variety of indicators of team effectiveness. Therefore, future research might test whether collective prosocial motivation acts as an alternative meditator between CSR and collective OCB.

Forth, our model solely focuses on the mechanism through which firm-level CSR impacts collective OCB, but it does not include any moderators. Previous micro-level studies have identified certain individual characteristics as moderators of the relationship between CSR and OI at the individual level, such as moral identity (Wang et al., 2017), calling orientation (Hameed et al., 2016), and social and cultural orientations (Farooq et al., 2017). Thus, future research might incorporate some firm-level moderating variables, such as ethical climate (Shin, 2012) and cooperative norms (Shen and Benson, 2016), to further elucidate the strengths of the mediation relationships found in our study.

## Data Availability Statement

The datasets generated for this study are available on request to the corresponding author.

## Ethics Statement

The studies involving human participants were reviewed and approved by the Ethics Committee of School of Psychology at Beijing Normal University. The patients/participants provided their written informed consent to participate in this study.

## Author Contributions

X-HW wrote the sections of introduction, hypothesis development, and discussion. JY wrote the sections of Materials and Methods and Results. RC and BL designed the research, collected and analyzed the data.

## Conflict of Interest

The authors declare that the research was conducted in the absence of any commercial or financial relationships that could be construed as a potential conflict of interest.
